# A Periprosthetic Femoral Fracture with Characteristics of Atypical Femoral Fracture

**DOI:** 10.1155/2019/1275369

**Published:** 2019-07-16

**Authors:** Shuya Tanaka, Tomoaki Fukui, Keisuke Oe, Tomoyuki Matsumoto, Takehiko Matsushita, Shinya Hayashi, Teruya Kawamoto, Ryosuke Kuroda, Takahiro Niikura

**Affiliations:** Department of Orthopaedic Surgery, Kobe University Graduate School of Medicine, Kobe, Hyogo, Japan

## Abstract

Although the definition of atypical femoral fracture (AFF) excludes periprosthetic femoral fracture (PFF), the number of reports about PFF with characteristics of AFF is increasing. We present the case of such a fracture in this report. An 87-year-old woman who underwent bipolar hip arthroplasty for a femoral neck fracture 38 months prior reported left thigh pain with no history of trauma. Radiographs showed a simple transverse fracture at the level of the stem distal end with features of AFF: periosteal thickening of the lateral cortex, a medial spike, and a noncomminuted fracture. She presented other features resembling AFF: history of bisphosphonate use, prodromal symptoms, no associated trauma, and lateral bowing of the contralateral femur. The fracture showed nonunion after the initial osteosynthesis, and a revision surgery of the arthroplasty and osteosynthesis was performed. Nine months after the surgery, bony union was achieved and she regained the ability to walk. It is supposed that the fracture was influenced by a stress force related to implants and lateral bowing concentrating on the fracture site as a mechanical factor in addition to bisphosphonates as a biological factor. It would be important to recognize that AFF could occur at the peri-implant location, and early detection and treatment are essential.

## 1. Introduction

The incidence of hip arthroplasty for degenerative diseases, such as hip osteoarthritis or femoral neck fractures, is continuously increasing because of the aging of the population; accordingly, the cases of periprosthetic femoral fracture (PFF) are also increasing [1]. The American Society for Bone and Mineral Research published the Task Force reports of atypical femoral fractures (AFFs) in 2010 [2] and 2014 [3]. Although PFF was described as one of the exclusion criteria in the definition of AFF, both in 2010 and 2014, several reports about PFFs with features of AFF have been recently published [4–17]. In the current case report, we present a case with aspects of both PFF and AFF that we treated.

## 2. Case Report

**Figure 1:**
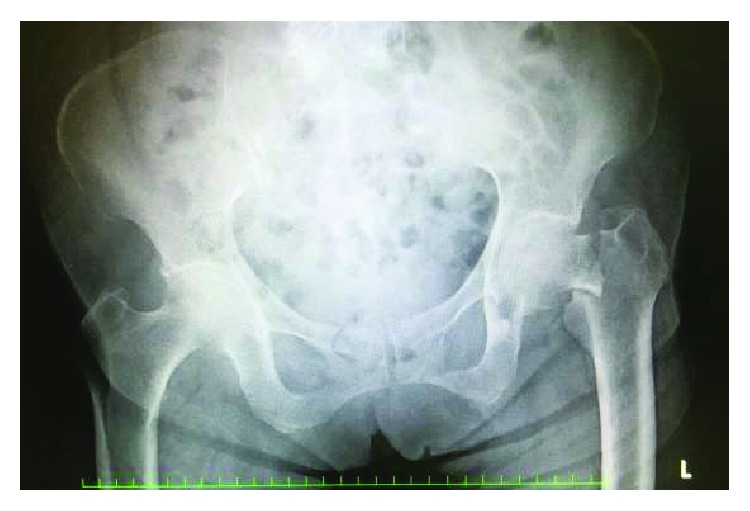
Radiograph of the initial left femoral neck fracture.

**Figure 2:**
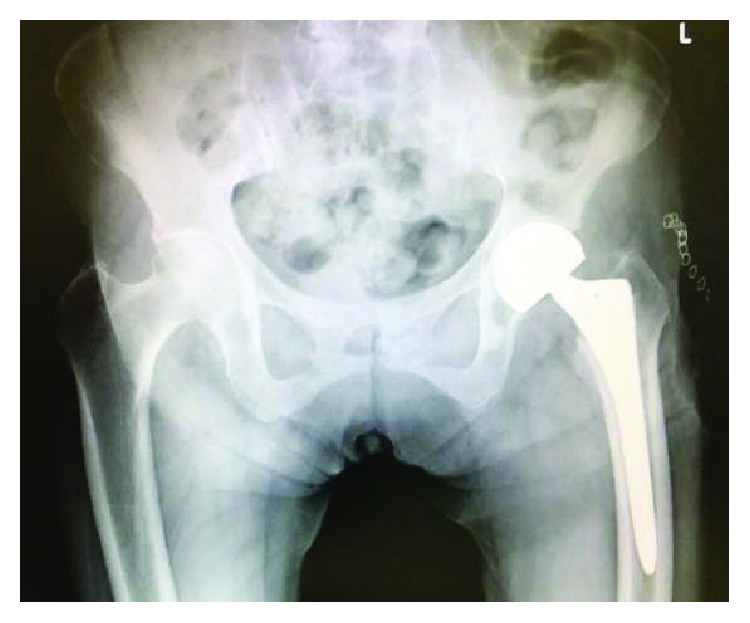
Radiograph just after bipolar hip arthroplasty.

**Figure 3:**
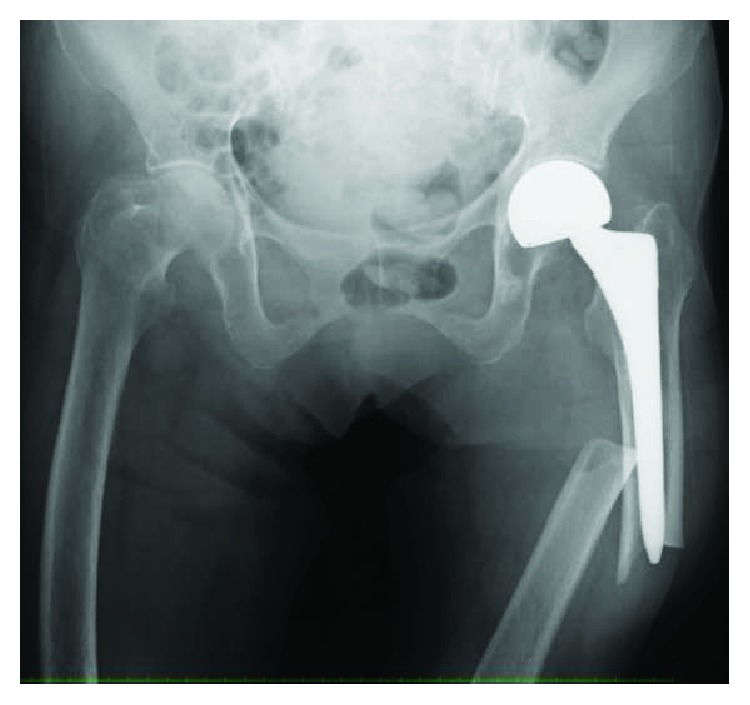
Radiograph shows a periprosthetic femoral fracture. A transverse fracture line around the stem tip progresses across the femur.

**Figure 4:**
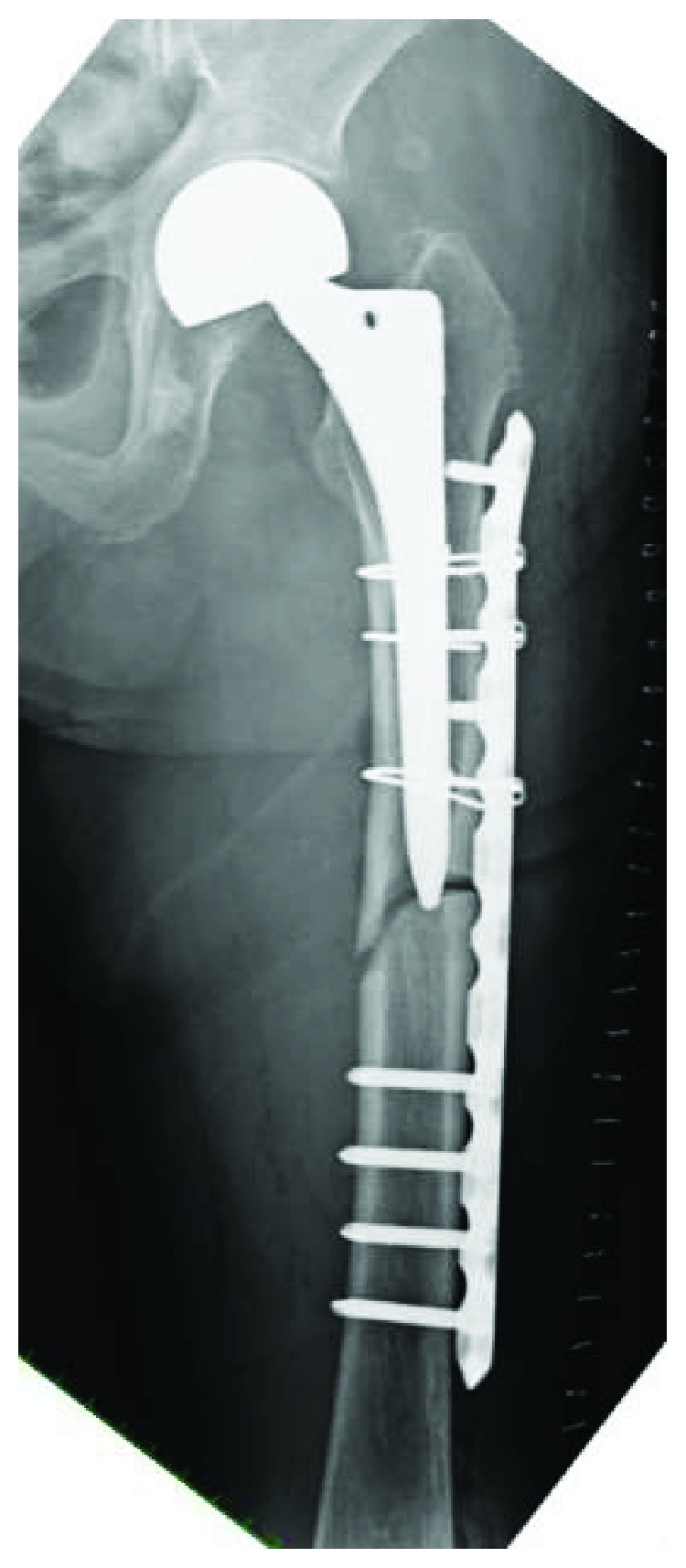
Radiograph just after osteosynthesis with a locking plate on the lateral side of the femur.

**Figure 5:**
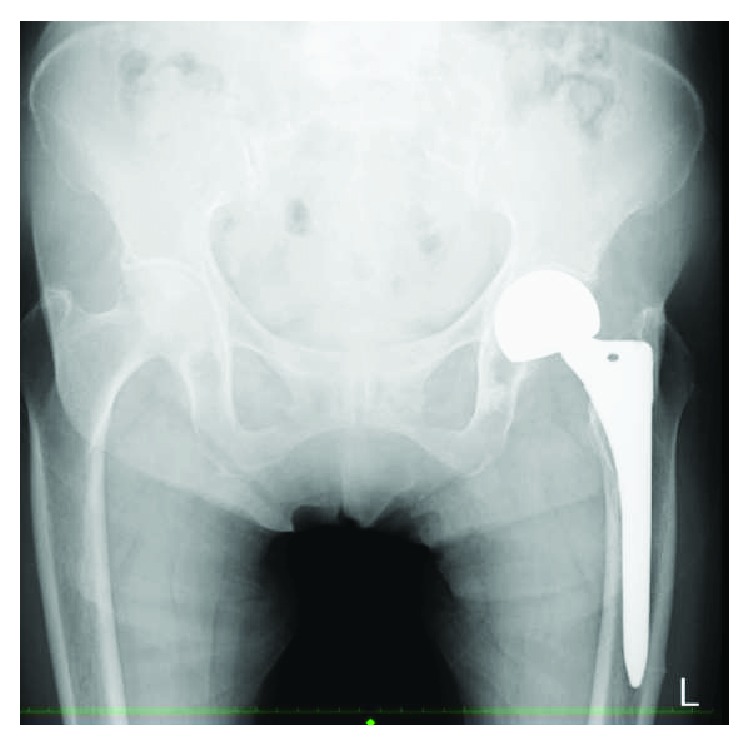
Radiograph 2 months before the periprosthetic fracture shows localized periosteal thickening of the lateral cortex at stem tip level.

An 87-year-old woman with left femoral nonunion following periprosthetic fracture after bipolar hip arthroplasty was referred to our department. She had undergone bipolar arthroplasty for left femoral neck fracture (Figures 1 and 2) and had been treated for osteoporosis with alendronate for 27 months after the bipolar arthroplasty. Her medical history included rheumatoid arthritis since the age of 60 years and diabetes mellitus diagnosed at the age of 83 years that was being treated using voglibose. She noticed thigh pain 36 months after the operation, which worsened 2 months later without any episode of injury. According to her, she felt pain at first and then fell because of the pain. A periprosthetic simple transverse fracture at the level of the distal end of the stem was found (Figure 3). Internal fixation with a locking plate was performed at the hospital (Figure 4). The remaining gap between the fragments was evident. To promote fracture healing, low-intensity pulsed ultrasound and teriparatide administration were started six days and two months after the surgery, respectively. Despite these treatments, the fracture did not heal in nine months; she was therefore referred to our department. The retrospective radiographic analysis revealed that a periosteal thickening of the lateral cortex at the stem tip level had existed at least two months before the fracture (Figure 5).

**Figure 6:**
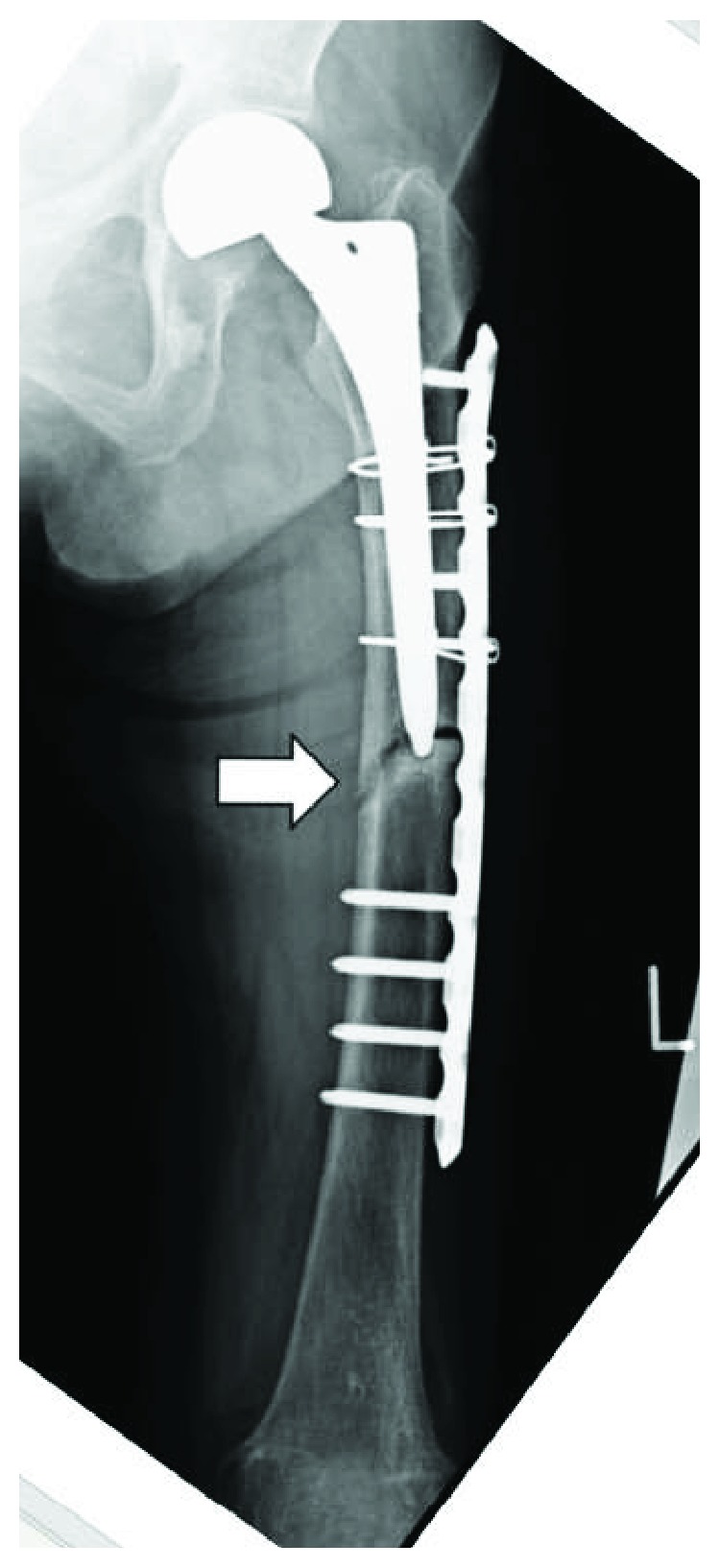
Radiograph 9 months after the osteosynthesis showing nonunion at the site of the fracture.

**Figure 7:**
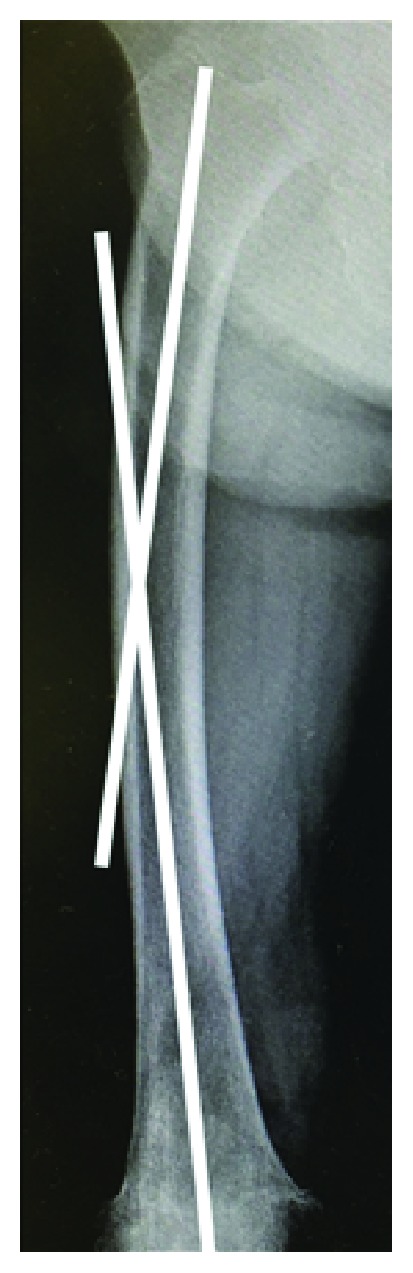
Radiograph of the right (contralateral) femur. Femoral bowing was measured as the angulation between the proximal and distal quarters of the femoral diaphysis. It was 13 degrees in this case.

On admission to our hospital, she could hardly walk due to the left thigh pain. Radiographs showed a nonunion fracture with osteosclerotic changes in the left femoral shaft at the level of the stem tip (Figure 6). Radiographs of her contralateral femur showed lateral bowing but no features of AFF (Figure 7).

**Figure 8:**
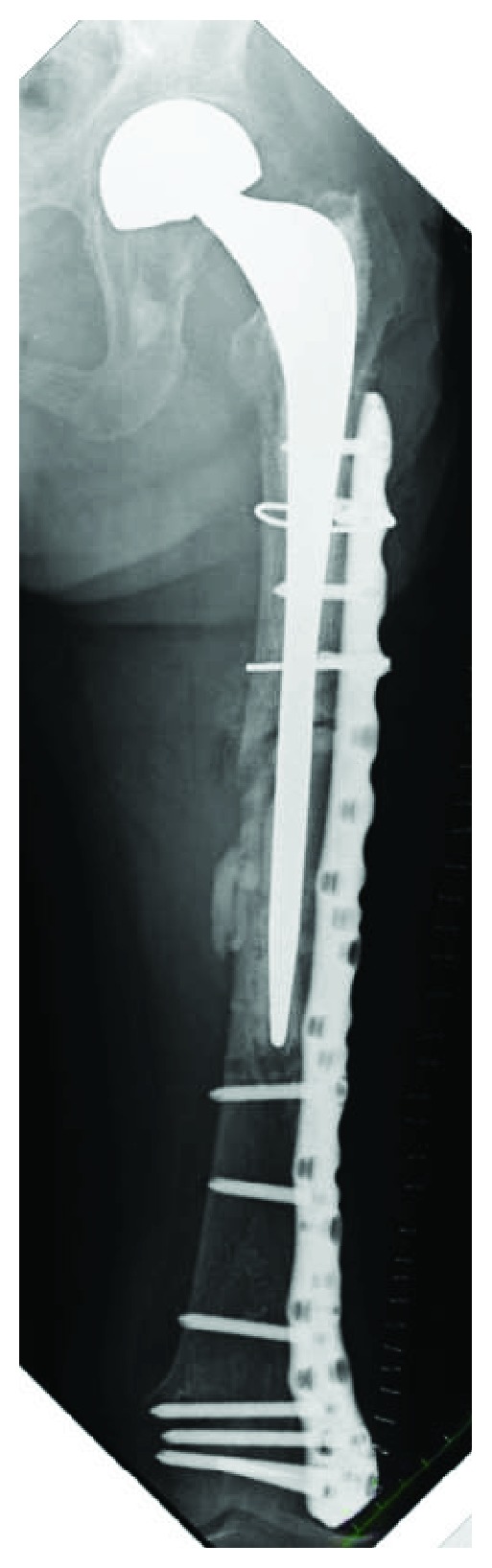
Radiograph just after a revision surgery with a longer stem with a cemented technique and a locking plate on the lateral side of the distal femur.

The implant was replaced with a longer stem (Exeter; Stryker, Tokyo, Japan) using the cemented technique. The implant of bipolar arthroplasty was not loosened. Osteosynthesis was performed with a locking plate for the distal femur (NCB Periprosthetic Femur Plate; Zimmer Biomet, Tokyo, Japan) and a cable system (Figure 8).

**Figure 9:**
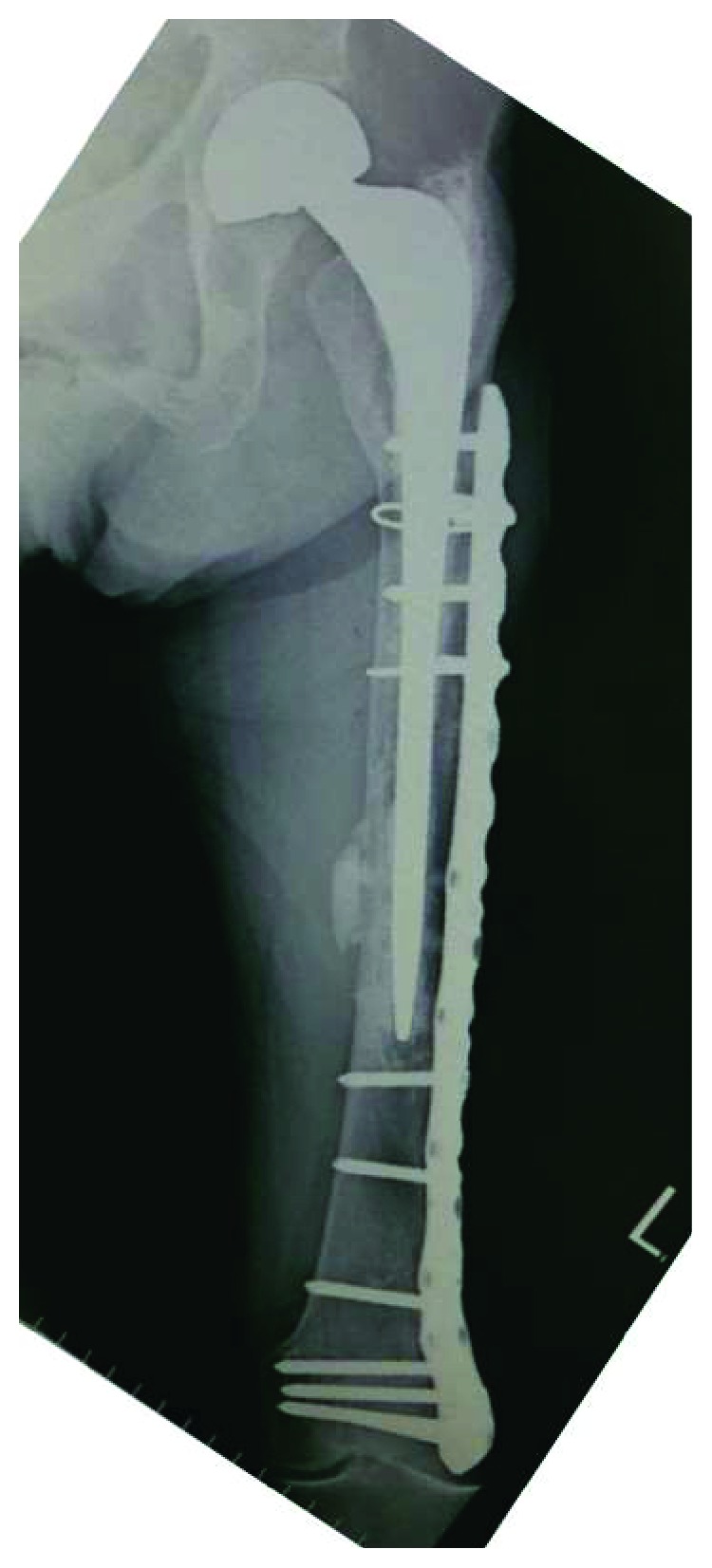
Radiograph 2 years after the revision surgery showing bone union achieved.

She regained the ability to stand up and walk without either a cane or a walker for short distances with no pain, as seen on the latest follow-up performed two years after the last surgery. Bony union was successfully achieved, and no issues were seen with the hemiarthroplasty (Figure 9).

## 3. Discussion

As mentioned above, PFF is currently excluded from the definition of AFF on the basis of the ASBMR task force reports [2, 3]. On the other hand, PFFs with characteristics of AFF exist and the number of reports about such cases is increasing (Table 1) [4–17]. Robinson et al. reported a retrospective multicenter case study of 196 AFFs in 2016 [17]. The patient population contained 21 cases (11%) of PFF, which was described as a periprosthetic AFF. Comparison between AFF and periprosthetic AFF showed a clinical difference in time to union, about five and eight months on average, respectively. In another paper, Corten et al. reported a mean union time of 6.4 months for typical PFFs [18]. These indicate that periprosthetic AFF might require a longer time for union than AFF and PFF. They noted that it was difficult to determine what factors were important to shorten union time from studying a small number of cases; therefore, larger-scale studies are required to address the question.

It is hypothesized that AFF would be affected not only by biological factors such as bisphosphonates or proton pump inhibitors but also by mechanical factors such as physical stress against the lateral cortex of the femur [10]. Lateral bowing of the femur in the frontal plane is indicated as one of the mechanical factors [19, 20]. Oh et al. reported that 6 of 12 cases of low-energy femoral shaft fractures were not treated with bisphosphonates at all. They proposed that stress fractures associated with a femoral shaft bowing deformity existed and should be recognized as another cause of AFFs [21]. Yoo et al. described that the diaphyseal AFFs were more frequent than subtrochanteric AFFs, if the lateral bowing angle was greater than 5.25 degrees [22]. Kharazmi et al. proposed lateral plating that could work as tension band plating against tensile forces in the lateral side of the bowing femur as prophylactic treatment for incomplete AFF and reported its effectiveness. This might prove that lateral bowing would be involved in the development of AFF [23].

Periprosthetic AFF should also be influenced by these two factors. That is, a stress force caused by femoral bowing and/or implants concentrating on the fracture site as well as the use of bisphosphonates, which has a suppressive effect on bone turnover, could interfere with bone remodeling and healing of stress fractures and microfractures [24]. Roberts et al. described that short and/or an increased femoral bowing on a lateral roentgenogram are more likely to have anterior cortical impingement with an intramedullary nail [25]. The former factor of periprosthetic AFF, stress force, might be caused by a similar mechanism to that reported for femoral deformity and stress due to an implant. Considering the causative factor of the current periprosthetic AFF case, both biological factors and mechanical factors were likely related in combination. The duration of bisphosphonate use was only 11 months; therefore, the mechanical factor might have been the stronger influence compared to the biological factor. The contralateral femoral bowing angle of the current case was 13 degrees (Figure 7), and this case could be considered to have a predisposition to develop atypical fractures. We cannot assess the radiograph of the affected femur before the fracture; however, it is suggested that there was a lateral bowing of the femur on the affected side in the same manner as on the contralateral side.

AFF should be treated with internal fixation as an initial treatment even for nondisplaced fractures [26]. Egol et al. described that differences were seen in healing time between AFFs reduced anatomically and those reduced nonanatomically; anatomically reduced fractures heal approximately 3.7 months faster than those fixed in varus [27]. This means that we must be careful to reduce periprosthetic AFF as precisely as possible.

The reason the current fracture led to nonunion after the first osteosynthesis could be explained by the mechanical aspect. A nonnegligible gap between fragments after the first osteosynthesis can negatively affect bony union. As the revision surgery, we performed a replacement using a longer stem to penetrate the sclerotic nonunion site and added a longer locking plate to increase rotational stability and to enable a fixation of the whole femur to reduce the risk of secondary fracture. In addition, this plate allows polyaxial locking screw placement, which is advantageous for the fixation of periprosthetic fractures. This locking plate might contribute to the stability of the fracture site as tension band plating as described previously [23].

Increasing evidence indicates that there are periprosthetic fractures that have a pathology similar to that of AFFs. We must be vigilant and aware of this type of periprosthetic fractures.

## Figures and Tables

**Table 1:** The cases of periprosthetic fractures resembling AFF described in the literature. Bisphosphonates were used before the fracture in all cases.

	Age	Sex	Surgery	Complication
Sayed-Noor AS, 2009	78	F	Locking plate	No
Curtin BM, 2011	52	F	No	No
Curtin BM. 2011	85	F	No	No
Curtin BM, 2011	79	F	No	No
Cross MB, 2012	81	F	No	No
Chen F, 2012	81	F	Locking plate	No
Schaeffer JF, 2012	79	F	Longer stem revision	No
Reb CW, 2013	74	F	Longer stem revision	No
Bhattacharyya R, 2014	72	F	No	No
Niikura T, 2015	69	F	Locking plate	No
Lee KJ, 2015	43	F	Locking plate	Breakage of plate⇒adding another plate
Lee KJ, 2015	74	F	Locking plate	No
Lee KJ, 2015	86	F	Locking plate	No
Wakayama T, 2015	68	F	Locking plate	No
Woo SB, 2016	82	F	Locking plate	Breakage of plate ⇒Longer stem revision and adding another plate
Bottai V, 2017	77	F	Locking plate	Breakage of plate ⇒Revision to another plate
